# The Role of Epithelial Sodium Channel ENaC and the Apical Cl^-^/HCO_3_^-^ Exchanger Pendrin in Compensatory Salt Reabsorption in the Setting of Na-Cl Cotransporter (NCC) Inactivation

**DOI:** 10.1371/journal.pone.0150918

**Published:** 2016-03-10

**Authors:** Mina Patel-Chamberlin, Mujan Varasteh Kia, Jie Xu, Sharon Barone, Kamyar Zahedi, Manoocher Soleimani

**Affiliations:** 1 Center on Genetics of Transport and Epithelial Biology, University of Cincinnati, Cincinnati, Ohio, United States of America; 2 Research Services, Veterans Affairs Medical Center, Cincinnati, Ohio, United States of America; 3 Department of Medicine, University of Cincinnati, Cincinnati, Ohio, United States of America; University of Pittsburgh, School of Medicine, UNITED STATES

## Abstract

**Background:**

The absence of NCC does not cause significant salt wasting in NCC deficient mice under basal conditions. We hypothesized that ENaC and pendrin play important roles in compensatory salt absorption in the setting of NCC inactivation, and their inhibition and/or downregulation can cause significant salt wasting in NCC KO mice.

**Methods:**

WT and NCC KO mice were treated with a daily injection of either amiloride, an inhibitor of ENaC, or acetazolamide (ACTZ), a blocker of salt and bicarbonate reabsorption in the proximal tubule and an inhibitor of carbonic anhydrases in proximal tubule and intercalated cells, or a combination of acetazolamide plus amiloride for defined durations. Animals were subjected to daily balance studies. At the end of treatment, kidneys were harvested and examined. Blood samples were collected for electrolytes and acid base analysis.

**Results:**

Amiloride injection significantly increased the urine output (UO) in NCC KO mice (from 1.3 ml/day before to 2.5 ml/day after amiloride, p<0.03, n = 4) but caused only a slight change in UO in WT mice (p>0.05). The increase in UO in NCC KO mice was associated with a significant increase in sodium excretion (from 0.25 mmol/24 hrs at baseline to 0.35 mmol/24 hrs after amiloride injection, p<0.05, n = 4). Daily treatment with ACTZ for 6 days resulted in >80% reduction of kidney pendrin expression in both WT and NCC KO mice. However, ACTZ treatment noticeably increased urine output and salt excretion only in NCC KO mice (with urine output increasing from a baseline of 1.1 ml/day to 2.3 ml/day and sodium excretion increasing from 0.22 mmole/day before to 0.31 mmole/day after ACTZ) in NCC KO mice; both parameters were significantly higher than in WT mice. Western blot analysis demonstrated significant enhancement in ENaC expression in medulla and cortex of NCC KO and WT mice in response to ACTZ injection for 6 days, and treatment with amiloride in ACTZ-pretreated mice caused a robust increase in salt excretion in both NCC KO and WT mice. Pendrin KO mice did not display a significant increase in urine output or salt excretion after treatment with amiloride or ACTZ.

**Conclusion:**

1. ENaC plays an important role in salt reabsorption in NCC KO mice. 2. NCC contributes to compensatory salt reabsorption in the setting of carbonic anhydrase inhibition, which is associated with increased delivery of salt from the proximal tubule and the down regulation of pendrin. 3. ENaC is upregulated by ACTZ treatment and its inhibition by amiloride causes significant diuresis in NCC KO and WT mice. Despite being considered mild agents individually, we propose that the combination of acetazolamide and amiloride in the setting of NCC inhibition (i.e., hydrochlorothiazide) will be a powerful diuretic regimen.

## Introduction

Kidney is the major organ responsible for maintaining electrolyte balance and acid-base homeostasis through the absorption of NaCl and secretion of acid or base equivalents in various nephron segments [1–21**]**. The bulk of Na^+^, Cl^−^, and HCO_3_^−^ is reabsorbed in the proximal tubule via an apical Na^+^/H^+^ exchanger and a Cl^−^/base exchanger acting in parallel [1–4]. Salt absorption in the thick ascending limb (TAL) and the distal convoluted tubules (DCT) is primarily mediated via NKCC2 (apical Na-K-Cl cotransporter) and NCC (Na-Cl cotransporter), respectively [5–8]. The collecting duct (CD) and the connecting tubule (CNT) are involved in the fine-tuning of acid-base transport and electrolyte and fluid balance. The sodium absorption in the CNT and CCD (cortical collecting duct) is mediated via the epithelial sodium channel ENaC in principal cells, whereas chloride is absorbed primarily via pendrin, an apical Cl^−^/HCO_3_^-^ exchanger expressed in non A-intercalated cells [9–16]. Recent studies suggest that the sodium-dependent chloride bicarbonate exchanger (NDCBE) also plays a role in salt absorption in CCD [17, 18].

Based on its dual function of bicarbonate secretion and chloride reabsorption, it was originally thought that pendrin plays important roles in acid base regulation and electrolyte homeostasis under basal conditions. Mice with the genetic deletion of pendrin or humans with inactivating mutations in PDS gene, however, do not display excessive salt and fluid wasting or altered blood pressure under baseline conditions [22, 23]. Similarly, mice with the genetic ablation of NCC do not display excessive salt wasting under baseline conditions [24].

Very recent reports have unmasked the basis of incongruity between the mild phenotype in NCC KO- or pendrin KO mice, and the role of pendrin and NCC as important players in salt reabsorption in the distal tubule. These studies demonstrate that pendrin and NCC cross compensate for the loss of each other, therefore masking the role that each transporter plays in salt reabsorption under baseline conditions [25]. The purpose of the current studies was to test the effect of inhibition of ENaC, as a collaborating partner of pendrin, or the down regulation of pendrin on salt excretion in NCC KO mice.

## Methods

### Animal models

Wild type (WT), NCC KO and pendrin KO mice, on C57BL/6 background, were utilized for these studies. Details of generation of pendrin (Slc26a4) null mice and the thiazide sensitive Na-Cl cotransporter (NCC; Slc12a3) null mice (generous gifts from Dr. Gary Shull) have been reported before [23, 24]. The Institutional Animal Care and Use Committee (IACUC) at the University of Cincinnati approved these studies. All animal handlers were IACUC-trained. Animals had access to food and water *ad libitum*, were housed in humidity-, temperature-, and light/dark- controlled rooms, and were inspected daily. Animals were euthanized with the use of excess anesthetics (pentobarbital sodium) according to institutional guidelines and approved protocols.

### Experimental protocols

For experiments, mice were injected with 5mg/kg/day of amiloride, a specific inhibitor of ENaC for 5 days, or 100mg/kg body weight/day of acetazolamide (ACTZ), a carbonic anhydrase inhibitor, for 6 days [26–28]. ACTZ was dissolved in 1:4 solution of DMSO:Sunflower Seed Oil. Animals were subjected to balance studies. At the end of treatments, kidneys were harvested and processed for RNA and protein extraction. Blood samples were collected for electrolytes and acid base balance measurement. In separate set of experiments, WT and pendrin KO mice were treated with either amiloride (n = 5) for 5 days or with ACTZ for 6 days (n = 5). In additional set of experiments, WT and NCC KO mice were pretreated with ACTZ for 6 days and then received acetazolamide plus amiloride for 3 more days.

### Tail DNA genotyping

#### NCC KO genotyping

NCC primers: KO forward, 5^’^AGG GTC AAG GGC ACG GTT GGC 3^’^; KO reverse, 5^’^GGT AAA GGG AGC GGG TCC GAG G 3^’^; KO rev pk, 5^’^GCA TGC TCC AGA CTG CCT TG 3^’^. **PCR Conditions**: 1, 94°C 2 min 1 cycle; 2, 94°C 30 seconds 68°C 30 seconds, repeat for 35 cycle, **68**°C 3min; end and hold at 4°C.

#### Pendrin KO genotyping

The primers for pendrin genotyping were the same as those described in our original paper reporting the generation of pendrin KO mice [23] and are as follows: the first set of pendrin specific oligos: PDS A2, GGC AGG CAA GCA TTC TAC CAC TAA G; PDS F7, GGA ACT TCG CTA GAC TAG TAC GCG TG exclusively amplified a 1.8-kb (PDS KO) fragment of the ablated PDS gene. The second set of pendrin oligos: PDS-A2-1, GCA GGC AAG CAT TCT ACC AC; PDS-3-S, AGG TAA GAT GCT GCT GGA TAG G specifically amplified a 1.9-kb fragment of the WT PDS gene. The PCR conditions for both reactions were as follows: initial denaturation for 2 min at 94°C; followed by annealing, 35 cycles at 94°C for 30 s; extension, 65°C for 30 s; and final extension, 68°C for 2 min. Products of the two PCR reactions were size-fractionated. The presence of the 1.8- and 1.9-kb band was indicative of the mutant and wild type alleles, respectively.

### Antibodies

Pendrin antibodies were generated in our laboratory as described [23]. The polyclonal antibodies against ENaC subunits (α, β γ) were purchased from StressMarq (#SPC-402D, 404D and 505D), Victoria, BC.

### Western blot analysis

Membrane proteins were prepared from mouse kidneys as previously described [23, 25]. Membrane proteins were size-fractionated by SDS/PAGE (40μg/lane) and transferred to nitrocellulose membrane. Western blot analyses were performed using anti-ENaC antibodies. Appropriate horseradish peroxidase-conjugated IgGs (Thermo Scientific, Rockford, IL) were used as secondary antibodies. The bands were visualized by chemiluminescence method (Invitrogen, Carlsbad, CA), captured on light-sensitive imaging film (Denville Scientific Inc, Metuchen, NJ) and quantitated by densitometry.

### Immunofluorescence labeling studies

Animals were euthanized with an overdose of pentobarbital sodium and perfused through the left ventricle with 0.9% saline followed by cold 4% paraformaldehyde in 0.1 M sodium phosphate buffer (pH 7.4). Kidneys were removed, cut in tissue blocks, and fixed in formaldehyde solution overnight at 4°C. The tissue was either frozen on dry ice or fixed in paraffin, and 6-μm sections were cut with a cryostat and stored until used. Single-immunofluorescence labeling with pendrin antibodies was performed as described [23, 25].

### RNA isolation and Northern hybridization

Total cellular RNA was extracted from kidneys, according to established methods, quantitated spectrophotometrically; and stored at -80°C. Hybridization was performed according to established protocols [11, 19]. Gene specific DNA fragments were generated by RT-PCR and used as specific probes for Northern blot hybridization. For pendrin, a fragment encoding nucleotides 1883–2217 from a mouse cDNA was generated from mouse kidney and used. For ENaC subunits, PCR-amplified isoform specific fragments were prepared and used as specific probes for subunits α (nucleotides 1,197–1,890), β (nucleotides 1,012–1,848), and γ (nucleotides 135–790). Each Northern blot hybridization was performed at least on four different animals.

### Quantitative RT-PCR for analysis of NKCC2 isotype expression profile

Total RNA (1μg) isolated from kidneys of WT and pendrin/NCC dKO mice was used for first strand DNA synthesis. Real-time PCR for quantitation of NKCC2 isoforms and glycerol phosphate dehydrogenase (GAPDH) was performed using the SYBR® Green Real-Time PCR Master Mix (Life Technologies). The following oligonucleotide primers that were previously utilized for qRT-PCR analysis of NKCC2 and its isotype specific transcripts were used: NKCC2 antisense primer, 5’-CTA AGC TCC GGG AAA TCA GGT A-3’; NKCC2-A antisense primer, 5’-ccc agt gat aga ggt tac cat ggt-3’; NKCC2-B antisense primer, 5’-gac aaa cct gtg atg gct gtc a-3’; NKCC2-F antisense primer, 5’-aca act acg ctc agg cca atg-3’; NKCC2 sense primer, 5’-gcc tct cct gga ttg tag gag aa-3’. NKCC2 isoform mRNA expression results were normalized against GAPDH mRNA expression of the corresponding samples. The final results were expressed as fold change in the expression of each NKCC2 isotype in NCC KO compared to WT mice.

### Urine and serum electrolytes analysis

Mice were housed in metabolic cages and had free access to rodent chow and water. Food intake, water intake, and urine volume were measured daily. Urine was collected under mineral oil. Urine chloride and sodium concentrations were measured using a digital chloridometer (HBI Haake Buchler Instruments, Inc.). Serum concentration of Na^+^, K^+^, Ca^++^, and HCO_3_^-^ were measured using i-STAT^R^-1 analyzer with i-STAT EG7+ cartridges (Abbott Laboratories, Abbott Park, IL).

### Statistical analysis

The results for urine volume and salt excretion are presented as means ± SE. Statistical significance between wild-type and NCC KO or between WT and pendrin KO mice was determined by Student's unpaired t- test or ANOVA and P<0.05 was considered significant.

## Results

### Expression of ENaC in kidneys of NCC KO mice

**Fig 1:**
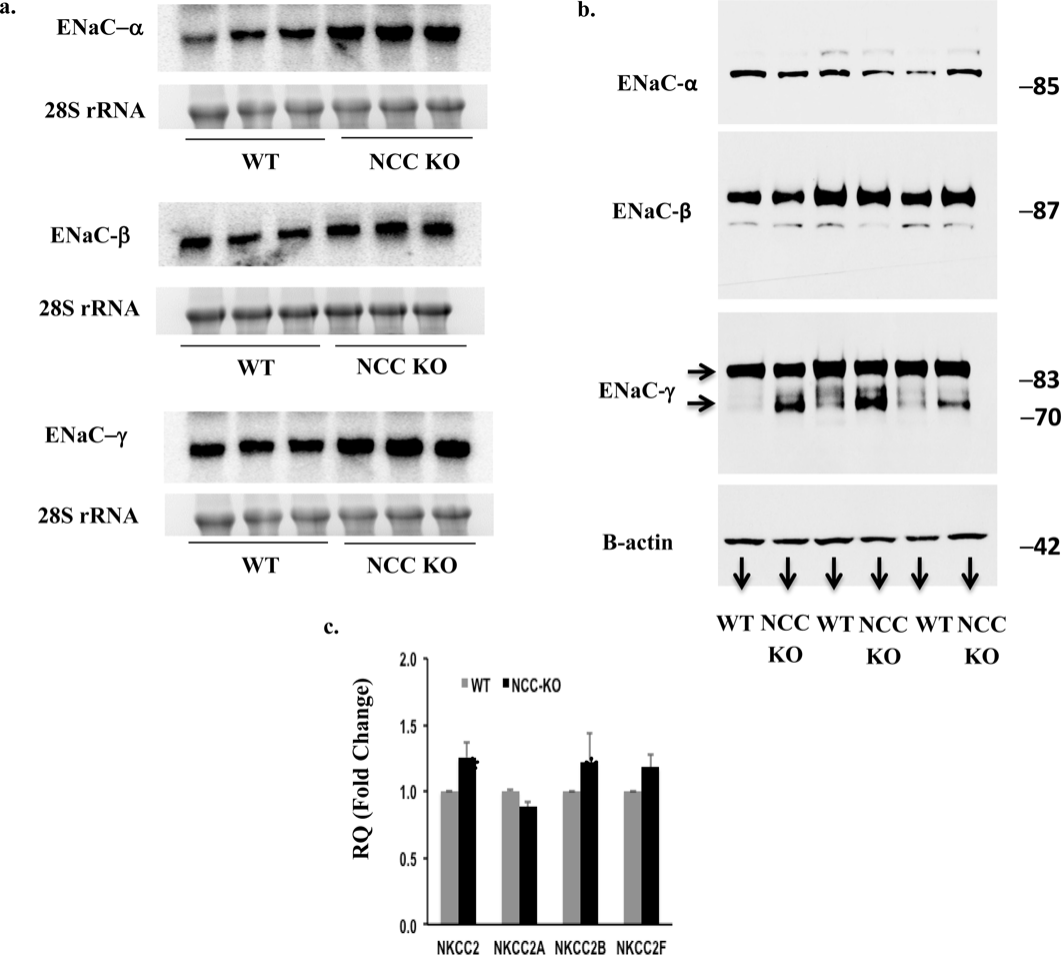
Expression of ENaC in kidneys of WT and NCC KO mice. Expression of ENaC subunits by (a) Northern hybridization and (b). Western blot in kidneys of WT and NCC KO mice. (a) Expression levels of all 3 subunits increased by Northern hybridization, with α, β and γ subunits increasing by 110, 80 and 70%, respectively, vs. wt littermates. Western blots show significant increase in the cleaved ENaC γ subunit in NCC KO mice, consistent with published reports (29). (b) The abundance of cleaved ENaC γ subunit form increased by ~420% in NCC KO mice. The full length bands for α, β and γ subunits are shown, which did not show significant changes vs. WT mice. c. Expression of NKCC2 isotypes in WT and NCC KO mice.

The northern hybridizations and western blots in Fig 1 depict the expression of ENaC subunits in kidneys of WT and NCC KO mice. Northern hybridizations show significant upregulation of mRNA encoding ENaCα, βand γ subunits in kidneys of NCC KO mice (Fig 1A). The mRNA expression levels forα, β and γ subunits, when normalized for 28S rRNA intensity, increased by 110, 80 and 70%, respectively, in kidneys of NCC null mice vs. wild type mice (p<0.02, <0.05 and <0.05, vs. WT). The analysis of Western blots by densitometric scanning demonstrated the upregulation of cleaved form (70 kDa) of ENaC γ subunit in kidneys of NCC KO mice (Fig 1B), with the cleaved band increasing by ~420% in NCC KO mice (p<0.02). Expression levels of full length ENaCα, β and γ subunits remained unchanged vs. WT mice (Fig 1B), confirming the published reports [29].

### Expression of NKCC2 in kidneys of NCC KO mice

The expression levels of NKCC2 and its isotypes in the kidneys of WT and NCC-KO mice were compared using qRT-PCR analysis, as described in Method Section. The results are depicted in Fig 1C and indicate a mild but significant increment in the expression of total NKCC2 and NKCC2 B isotype.

### Effect of amiloride on urine output and water intake in NCC KO and WT mice

**Fig 2:**
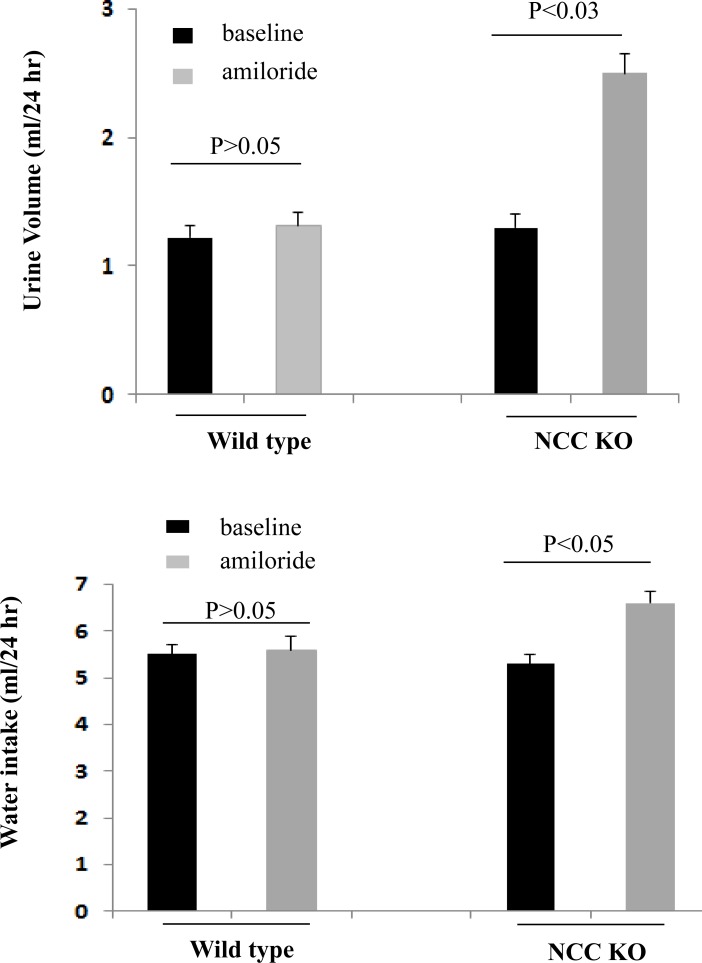
Effect of amiloride on urine output and water intake in WT and NCC KO mice. After acclimation in metabolic cages, wild type and NCC KO mice were treated with daily intraperitoneal (IP) injection of amiloride. Water intake and urine output were collected daily for 5 days. Water intake (a) and urine output (b) on day 3 and beyond were comparable.

Mice in metabolic cages were treated with daily intraperitoneal (IP) injection of amiloride for 5 days. Urine output was collected daily. For the analysis, urine output collections on day 3 of amiloride injection were compared between WT and NCC KO mice. As shown in Fig 2A, urine output almost doubled in NCC KO mice after amiloride injection (baseline urine output was 1.30 ml and increased to 2.5 ml/24 hrs after amiloride injection). Urine output in WT mice did not change significantly after amiloride injection (baseline urine output was 1.22 ml per day, and was increased to 1.32 ml per/24 hrs after injection). Water intake was higher after amiloride in NCC KO mice but not in WT mice (Fig 2B).

### Effect of amiloride on sodium excretion in WT and NCC KO mice

**Fig 3:**
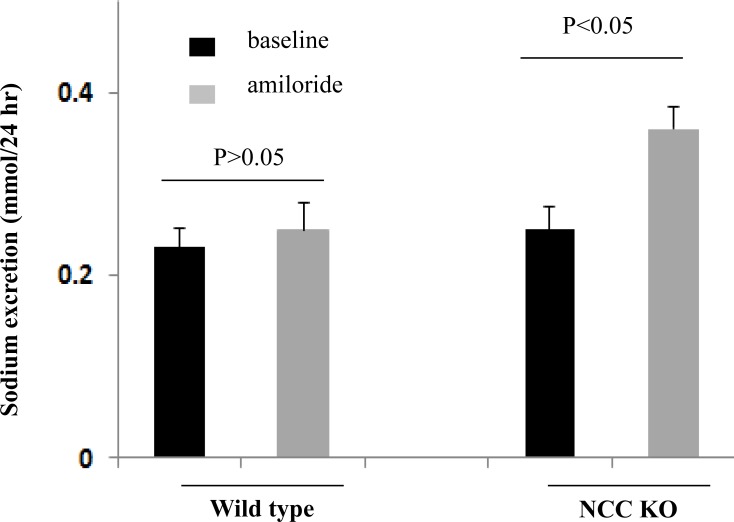
Effect of amiloride on sodium excretion in WT and NCC KO mice. Daily sodium excretion before and after treatment with amiloride is shown in WT and NCC KO mice. Sodium excretion on day 3 and beyond were comparable.

As shown in Fig 3, sodium excretion in NCC KO mice was significantly increased after amiloride injection (from 0.25 mmol/24 hrs at baseline and 0.35 mmol/24 hrs after amiloride injection, p<0.05) but remained unchanged in WT littermates.

### Enhanced expression of pendrin in kidneys of NCC KO mice

**Fig 4:**
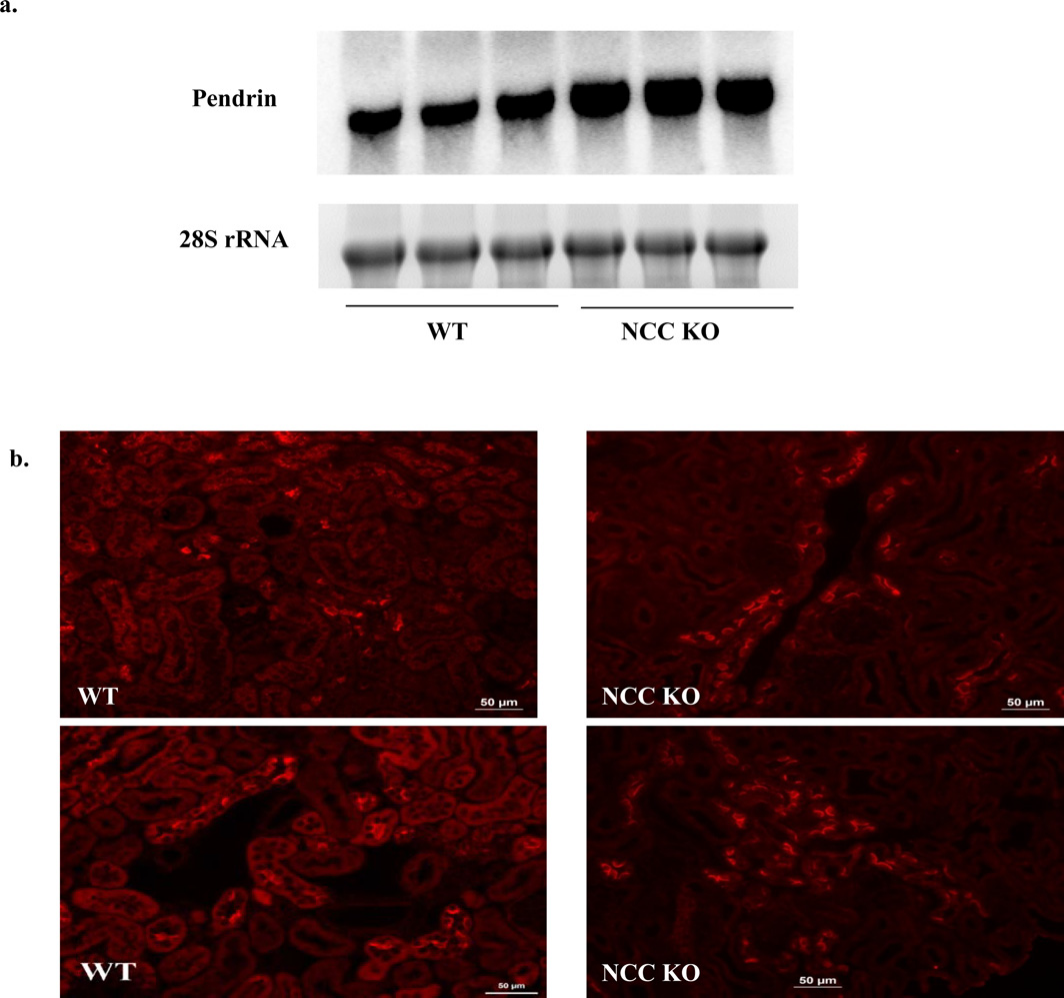
Expression of pendrin in kidneys of WT and NCC KO mice. Expression of pendrin by (a) Northern hybridization and (b) Immunofluorescence labeling in kidneys of WT and NCC KO mice. Northern hybridizations showed significant increase in pendrin expression in NCC KO mice vs. WT littermates (with expression of pendrin increasing by 130% vs. wt littermates, p<0.03). Immunofluorescence labeling showed significant increase in the number of pendrin positive cells in kidneys of NCC KO mice vs. WT littermates (p<0.01 vs. WT).

Fig 4 shows the expression of pendrin in kidneys of NCC KO and WT, as determined by northern hybridization (a) and immunofluorescence labeling (b). As indicated, mRNA expression of pendrin is significantly increased in kidneys of NCC KO mice compared to WT type (Fig 4A). Immunofluorescence labeling studies corroborated the upregulation of pendrin in kidneys of NCC KO mice by indicating increased number of pendrin positive cells as well as enhanced apical rim labeling pattern compared to subapical and/or cytoplasmic labeling in control animals (Fig 4B). The number of pendrin-positive cells in 10 random microscopic fields (X400) was 94.60 +/- 8.4 in WT mice vs. 232.60 +/- 24.8 in NCC KO mice (p<0.01, n = 3 different animals/group).

### Effect of acetazolamide (ACTZ) on pendrin expression, urine output and salt excretion in NCC KO mice

**Fig 5:**
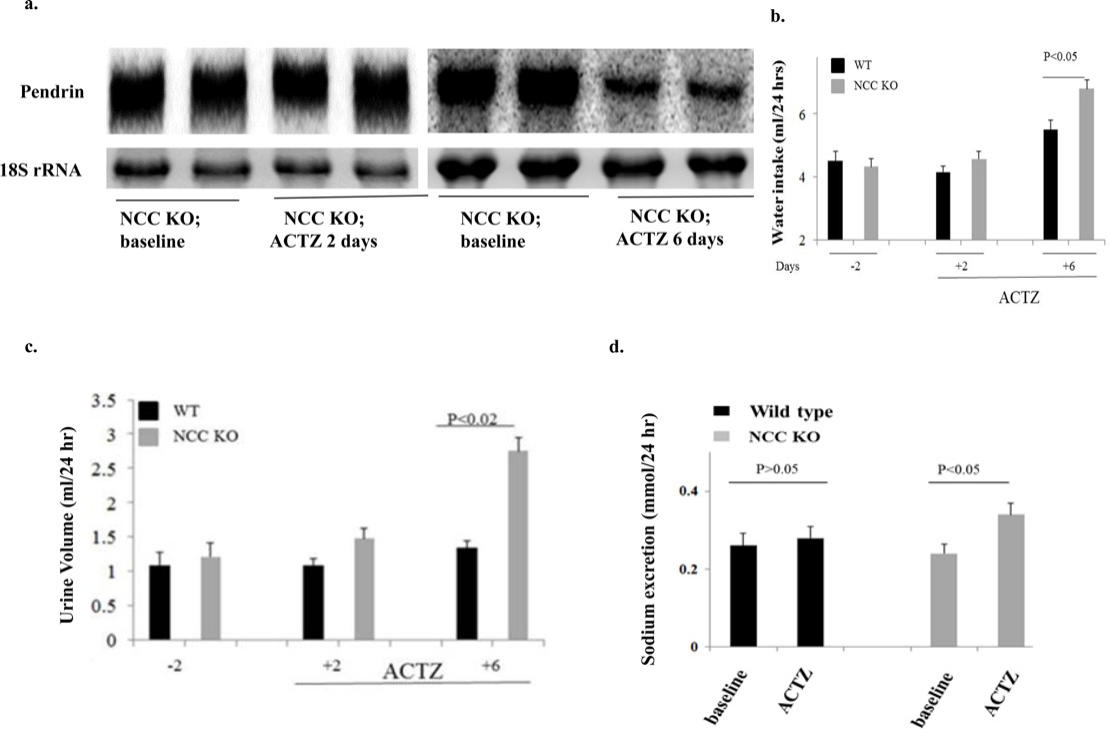
Effect of Acetazolamide on pendrin expression, urine output and water intake in WT and NCC KO mice. Fig 5a shows the expression of pendrin in WT and NCC KO mice treated with acetazolamide for 2 or 6 days (left and right panels). Fig 5b and 5c depicts the urine output (i) and water intake (ii) in WT and NCC KO mice treated with ACTZ. The increase in urine output by ACTZ in NCC KO mice correlated with a reduction in pendrin expression. Fig 5d demonstrates enhanced sodium excretion by ACTZ in NCC KO mice vs. WT animals.

Fig 5A shows a significant reduction in pendrin expression in NCC KO mice treated with acetazolamide for 6 days, as measured by Northern hybridization. The expression of pendrin was not significantly affected after 2 days of acetazolamide treatment (Fig 5A). We therefore compared water intake and urine output on day 2 (before pendrin downregulation) and day 6 (after pendrin downregulation). Fig 5B and 5C depicts the water intake and urine output in WT and NCC KO mice treated with ACTZ, and shows gradual increases in both the urine output and water intake in NCC KO by day 6 compared to WT mice. The increase in urine output by ACTZ in NCC KO mice correlated with a reduction in pendrin expression. Fig 5D demonstrates that the increase in urine output was associated with enhanced sodium excretion in NCC KO mice (0.24 mmol/day before and 0.34 mmol/day after 6 days of ACTZ injection, p<0.05).

### ENaC expression in cortex and medulla of NCC KO and WT mice treated with acetazolamide (ACTZ)

**Fig 6:**
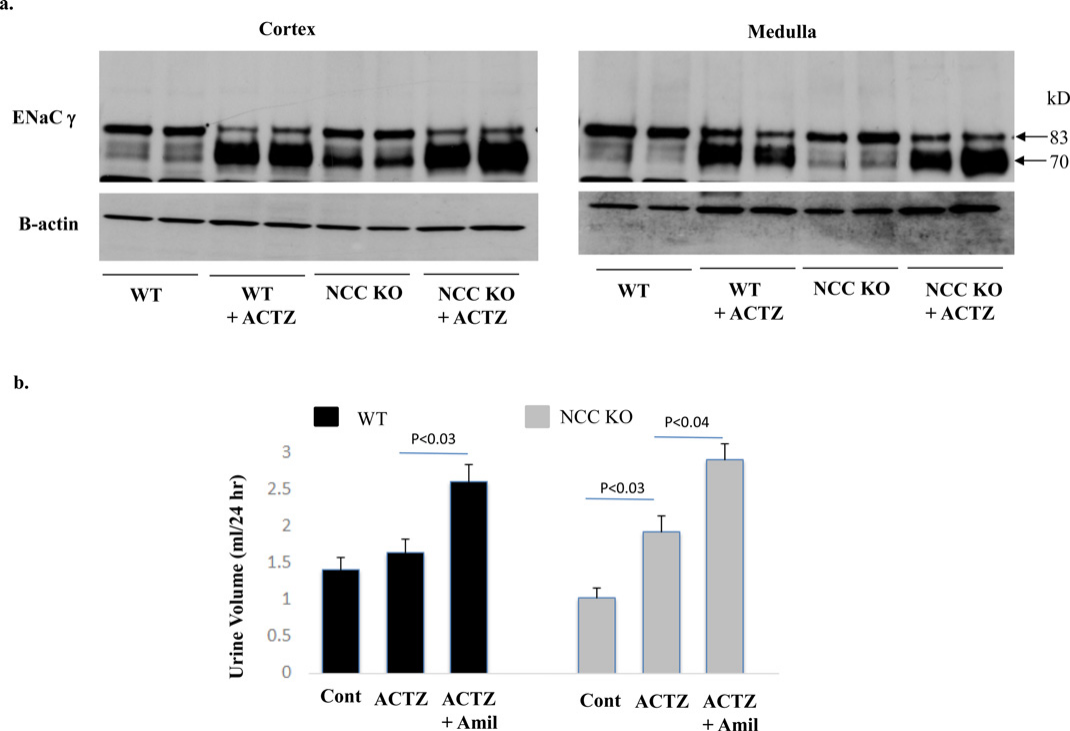
Effect of Acetazolamide on ENaC expression: Impact of amiloride on salt excretion in WT and NCC KO mice. Fig 6a shows the expression of ENaC in kidney medulla and cortex of WT and NCC KO mice treated with acetazolamide for 6 days (left and right panels). Fig 6b depicts the effect of amiloride treatment on salt excretion in WT and NCC KO mice pretreated with ACTZ for 6 days.

The expression of ENaC in microsomal membrane proteins isolated from cortex and medulla of NCC KO and WT mice treated with ACTZ for 6 days was examined. Fig 6A depicts the expression of ENaC γsubunit in kidneys of experimental animals. As indicated, the cleaved 70 kDa fragment, a reliable indicator of enhanced ENaC expression and activity, was significantly increased in both WT and NCC KO mice, with the NCC KO mice displaying a more robust increment in ENaC expression. These results strongly suggest that ENaC plays an important role in compensatory salt absorption in the collecting duct of ACTZ-treated mice.

### Effect of acetazolamide (ACTZ) plus amiloride on urine output and salt excretion in NCC KO mice

In the next series of studies, we examined the effect of amiloride on salt excretion in NCC KO vs. WT mice that were pretreated with ACTZ. Accordingly, both WT and NCC KO mice were treated with acetazolamide for 6 days, and then received acetazolamide plus amiloride for 3 more days. The results indicate that while salt excretion by ACTZ was more pronounced in NCC KO mice (Fig 6B, left panel), addition of amiloride increased salt excretion in both NCC KO and WT mice (Fig 6B, right panel). Fig 6B shows that whereas WT mice did not show a significant increment in salt excretion by ACTZ alone, they showed a robust increase in salt excretion when amiloride was added (Fig 6B).

### Effect of amiloride on salt excretion in pendrin KO mice

**Fig 7:**
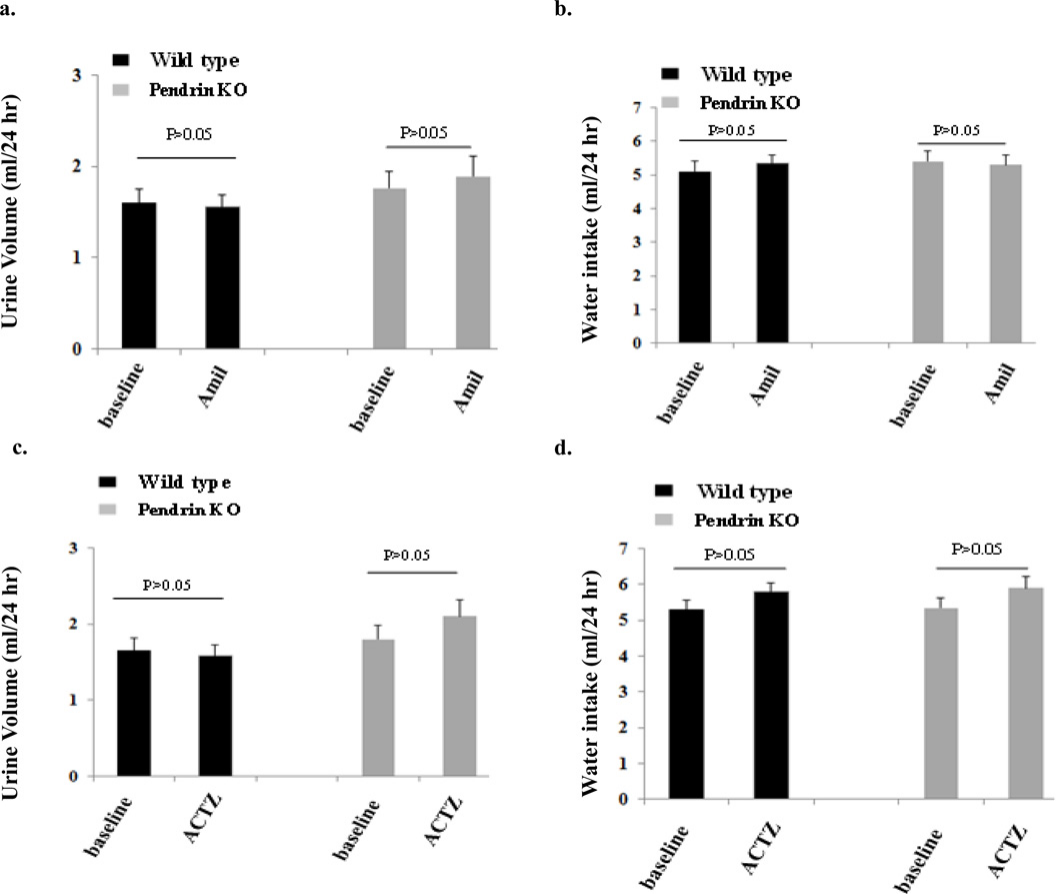
Effect of Amiloride and Acetazolamide on water and salt excretion in WT and pendrin KO mice. Fig 7a depicts urine output in pendrin KO mice compared to WT mice, before and after amiloride injection. Fig 7b shows water intake in the two genotypes at baseline and after amiloride injection. Fig 7c and 7d compares urine output and water intake in WT vs pendrin KO mice post ACTZ injection.

Fig 7A depicts urine output in pendrin KO mice compared to WT mice, before and after amiloride injection. As indicated, urine output in pendrin KO mice did not change significantly post amiloride injection and were comparable to that in WT mice. Similarly, water intake was comparable between the two genotypes at baseline or post amiloride injection (Fig 7B). The sodium excretion before amiloride was not significantly different in pendrin KO and WT mice (0.21 +/- 0.02 mmol/day in WT and 0.22 +/- 0.03 mmol/day in pendrin KO mice, p>0.05). Similarly, the sodium excretion after amiloride was comparable in pendrin KO mice vs. WT mice (0.23 +/- 0.02 mmol/day in WT and 0.0.24 +/- 0.03 mmol/day in pendrin KO mice, p>0.05).

### Effect of acetazolamide on urine output in pendrin KO mice

Fig 7C and 7D compares water intake and urine output in WT vs pendrin KO mice before and after ACTZ injection. As indicated, urine output in pendrin KO mice did not change after ACTZ injection for 6 days (Fig 7C). Similarly, ACTZ treatment did not have any significant effect on water intake in WT mice (Fig 7D). The sodium excretion under baseline conditions (before acetazolamide) was not significantly different in pendrin KO and WT mice (0.21 +/- 0.02 mmol/day in WT and 0.23 +/- 0.03 mmol/day in pendrin KO mice, p>0.05). Similarly, the sodium excretion was not significantly different in pendrin KO mice vs. WT mice after 6 days of acetazolamide (0.23 +/- 0.02 mmol/day in WT and 0.25 +/- 0.03 mmol/day in pendrin KO mice, p>0.05).

In the last series of experiments, we examined the serum electrolyte profile and acid base balance of NCC KO mice relative to WT mice. Our results indicate that serum Na^+^ concentrations were 139.7 +/- 2.1 mEq/l in WT and 142.7 +/- 0.9 in NCC KO mice (p>0.05, = 4). Serum K^+^ concentrations were 5.7 +/- 0.5 mEq/l in WT and 5.2 +/- 0.3 in NCC KO mice (p>0.05, n = 4). Serum HCO_3_- concentrations were 24.1 +/- 1.0 mEq/l in WT and 21.4 +/- 0.8 in NCC KO (p = 0.08, n = 4). Serum calcium concentrations were 0.90 +/- 0.004 mmol/l in WT and 1.01 +/- 0.004 in NCC KO mice (p<0.001, n = 4). Serum magnesium concentration which is known to be reduced in NCC KO mice [24] was not measured in our studies.

## Discussion

Approximately 5–7% of filtered sodium is reabsorbed in the distal convoluted tubule (DCT), predominantly through the thiazide sensitive Na-Cl cotransporter NCC (SLC12A3) [7, 8, 21, 30]. Inactivating mutations in NCC lead to Gitelman’s syndrome, an autosomal recessive disorder manifested with magnesium deficiency, potassium deficiency and mild salt wasting [31, 32]. While DCT is responsible for the absorption of a significant fraction of filtered salt, mutations in NCC, which is the main salt absorbing transporter in this segment only cause mild salt wasting in most affected individuals [31, 32]. Similar to humans, mice with NCC gene deletion show little to no salt salt wasting under baseline conditions [24].

The current studies demonstrate enhanced expression of ENaC subunits and pendrin in kidneys of NCC KO mice by northern hybridization and western blot and/or immunofluorescence labeling (Figs 1 and 4). Systemic acid base and electrolyte profile studies indicated a lower serum HCO_3_- concentration which did not achieve statistical significance (Results). Serum sodium and potassium concentrations were comparable in WT and NCC KO mice. There was a marked increase in serum calcium concentration in NCC KO mice, consistent with published reports indicating decreased calcium excretion in this genotype [24].

The results of our studies indicate that the inhibition of ENaC by amiloride causes a robust diuresis in NCC KO mice but not in wild type littermates (Figs 2 and 3). Our results further demonstrate that the down regulation of pendrin by acetazolamide (Fig 5) causes a strong diuresis only in NCC KO mice, with no significant effects in WT littermates (Fig 5). We further show that the impact of ENaC inhibition on salt excretion is unique to NCC KO mice and is not detected in pendrin KO mice (Fig 6). Similarly, acetazolamide did not cause significant diuresis in pendrin KO mice (Fig 6). The absence of a significant salt excretion in response to amiloride injection in WT mice under baseline conditions is in agreement with published reports [33].

The increased expression levels of ENaC γ subunit and pendrin are in agreement with published reports on the expression of these two proteins by western blots in NCC KO mice [29, 34]. Our results further indicate enhanced mRNA expression of ENaC subunits α, β and γ subunits and pendrin, suggesting that the upregulation of these two ion channels/transporters in kidneys of NCC KO mice is predominantly due to increased synthesis. Whether post translational modification of ENaC and pendrin through enhanced trafficking to the apical membrane plays any role in the activation of these two transporters remain speculative.

Neither Pendrin KO mice nor NCC KO mice exhibit any evidence of salt wasting under baseline conditions [22–24]. Both mutant models however show inability to retain salt and develop hypotension during salt restriction [24, 35]. These results have led investigators to conclude that NCC and pendrin are predominantly active during salt depletion or in response to excess aldosterone actions. Recent studies demonstrate that pendrin/NCC double KO mice develop profound salt wasting compared to WT or NCC KO mice, suggesting the important role of pendrin in compensatory salt absorption in the setting of NCC inactivation [25].

While the present studies support the view that pendrin and ENaC work in tandem to absorb salt in NCC KO mice, they do not exclude a role for NDCBE as a collaborating partner with pendrin. It has been suggested that NDCBE works in tandem with pendrin to mediate ENaC-independent, sodium absorption in NCC KO mice [17, 18]. The conclusive evidence with regard to an important role for NDCBE should come from studies aimed at NDCBE inactivation in the setting of NCC ablation. Such a mouse model (NCC/NDCBE double KO mice) should mimic the salt losing phenotype in NCC/pendrin double KO mice [25] if NDCBE and pendrin functionally collaborate with each other, as has been proposed [17, 18].

Carbonic anhydrase inhibitors such as acetazolamide are historically known to inhibit salt and bicarbonate reabsorption in the proximal tubule [36, 37]. However, they display mild diuretic effects, presumably due to compensatory salt absorption in the distal nephron. In addition to inhibiting the cytosolic carbonic anhydrase 2 (CAII) and the membrane-bound carbonic anhydrase 4 (CAIV) in the proximal tubule [36, 37], ACTZ is also known to inhibit the cytosolic CAII in intercalated cells of the collecting duct, causing their remodeling [38–40]. Published studies in mice lacking the CAII demonstrate a significant reduction in the number of intercalated cells and the downregulation of pendrin [39–41]. Indeed, mice with double deletion of CAII and NCC develop profound salt wasting and volume depletion due to the down regulation of pendrin in the setting of NCC inactivation [30].

Recent studies demonstrated a synergistic diuretic effect between ACTZ and hydrochlorothiazide (HCTZ), a specific inhibitor of NCC, in Sprague Dawley rats [42]. An interesting aspect of these studies was that when used individually, HCTZ and ACTZ caused mild diuresis, however, they caused massive diuresis when used together [42]. Acetazolamide was also shown to downregulate the expression of pendrin in rat [42]. These results strongly support the view that ACTZ inhibits pendrin, leaving NCC as the major salt absorbing transporter in the distal nephron in the setting of increased delivery of salt from the proximal tubule.

The current studies in NCC KO mice extend those observations by demonstrating that in the setting of NCC inactivation, ACTZ causes significant diuresis, indicating that NCC is the major salt absorbing transporter in the distal nephron in the setting of pendrin downregulation and increased delivery of salt from the proximal tubule. This conclusion is further supported by studies in pendrin KO mice that did not demonstrate any enhanced diuresis in response to ACTZ treatment (Results). We also found that the inhibition of ENaC in the setting of pendrin inactivation does not enhance diuresis. We interpret these latter studies to indicate that pendrin and ENaC work in tandem and inactivation of pendrin or inhibition of ENaC in the setting of a functional NCC (either in WT or in pendrin KO mice) does not cause excessive salt wasting.

The robust enhancement in the expression of ENaC in the cortex and medulla of WT and NCC KO mice pretreated with ACTZ and the vigorous diuresis by amiloride in these animals is intriguing. The significant diuresis by amiloride in WT and NCC KO mice suggests that the ENaC, which is upregulated in both genotypes, is functional and contributes to compensatory salt absorption. Given the fact that pendrin and NDCBE are only expressed in the kidney cortical collecting duct, these results suggest the activated ENaC in medullary collecting duct (Fig 6) is collaborating with another chloride absorbing transporter or pathway. Both Slc26a11 (KBAT), which is expressed on the apical membrane of intercalated cells in the CCD, OMCD and IMCD [19] as well as paracellular absorptive pathways are viable candidates as functional partners of ENaC, at least in OMCD and IMCD.

NKCC2 shows a mild but significant upregulation in the thick ascending limb of Henle in NCC KO mice (Fig 1). Our studies in NCC KO mice treated with furosemide, however, did not show any significant increase in urine output vs. WT mice (ASN 2011, abstract). It is plausible that ENaC and pendrin as well as Slc26a11 could contribute to compensatory salt absorption in the distal nephron in response to increased delivery of salt consequent to loop diuretics.

In conclusion, pendrin and ENaC play important roles in compensatory salt absorption in kidneys of NCC KO mice. Further, the downregulation of pendrin by ACTZ causes significant diuresis in the setting of NCC inactivation. Pretreatment with ACTZ robustly upregulates ENaC expression in both cortical and medullary collecting ducts, and treatment with amiloride causes significant diuresis in both NCC KO and WT mice. Despite being considered mild agents individually, we propose that the combination of acetazolamide and amiloride in the setting of NCC inhibition (i.e. with hydrochlorothiazide) is a powerful diuretic regimen.

## References

[1] Aronson PS, Giebisch G. Mechanisms of chloride transport in the proximal tubule. Am J Physiol. 273(2 Pt 2):F179–92, 1997 Review. 9277578 10.1152/ajprenal.1997.273.2.F179

[2] Bobulescu IA, Moe OW. Na+/H+ exchangers in renal regulation of acid-base balance. Semin Nephrol. 26(5):334–44, 2006 Review. 17071327 10.1016/j.semnephrol.2006.07.001 PMC2878276

[3] Wang X, Armando I, Upadhyay K, Pascua A, Jose PA. The regulation of proximal tubular salt transport in hypertension: an update. Curr Opin Nephrol Hypertens. 18(5):412–20, 2009 Review. 10.1097/MNH.0b013e32832f5775 19654544 PMC3722593

[4] Baum M. Developmental changes in proximal tubule NaCl transport. Pediatr Nephrol. 23(2):185–94, 2008 Review. 17684771 10.1007/s00467-007-0569-0

[5] Molony DA, Reeves WB, and Andreoli TE. Na^+^:K^+^:2Cl^−^cotransport and the thick ascending limb. Kidney Int 36: 418–426, 1989 2687569 10.1038/ki.1989.211

[6] Arroyo JP, Lagnaz D, Ronzaud C, Vázquez N, Ko BS, Moddes L, et al Nedd4-2 Modulates Renal Na+-Cl- Cotransporter via the Aldosterone-SGK1-Nedd4-2 Pathway. J Am Soc Nephrol. 22:1707–19, 2011 10.1681/ASN.2011020132 21852580 PMC3171941

[7] Kim GH, Masilamani S, Turner R, Mitchell C, Wade JB, Knepper MA. The thiazide-sensitive Na-Cl cotransporter is an aldosterone-induced protein. Proc Natl Acad Sci U S A. 95:14552–7, 1998 9826738 10.1073/pnas.95.24.14552 PMC24411

[8] Rozansky DJ, Cornwall T, Subramanya AR, Rogers S, Yang YF, David LL, et al Aldosterone mediates activation of the thiazide-sensitive Na-Cl cotransporter through an SGK1 and WNK4 signaling pathway. J Clin Invest. 119:2601–12, 2009 10.1172/JCI38323 19690383 PMC2735908

[9] Stockand JD. Vasopressin regulation of renal sodium excretion. Kidney Int. 78(9):849–56, 2010 10.1038/ki.2010.276 20736986

[10] Hughey RP, Kleyman TR. Functional cross talk between ENaC and pendrin. Am J Physiol Renal Physiol. 293(5):F1439–40, 2007 17855481 10.1152/ajprenal.00402.2007

[11] Soleimani M., Greeley T., Petrovic S., Wang Z., Amlal H., Kopp P., et al Pendrin: an apical Cl-/OH-/HCO3- exchanger in the kidney cortex. Am J Physiol Renal Physiol 280, F356–F364, 2001 11208611 10.1152/ajprenal.2001.280.2.F356

[12] Royaux I. E., Wall S. M., Karniski L. P., Everett L. A., Suzuki K., Knepper M. A., et al Pendrin, encoded by the Pendred syndrome gene, resides in the apical region of renal intercalated cells and mediates bicarbonate secretion. Proc Natl Acad Sci U S A 98, 4221–4226, 2001 11274445 10.1073/pnas.071516798 PMC31206

[13] Wall SM, Pech V. Pendrin and sodium channels: relevance to hypertension.J Nephrol.23 Suppl 16:S118–23, 2010. Review. 21170868

[14] Wagner CA, Mohebbi N, Capasso G, Geibel JP. The anion exchanger pendrin (SLC26A4) and renal acid-base homeostasis. Cell Physiol Biochem. 28(3):497–504, 2011 Review. 10.1159/000335111 22116363

[15] Pech V, Pham TD, Hong S, Weinstein AM, Spencer KB, Duke BJ, et al Pendrin modulates ENaC function by changing luminal HCO3-. J Am Soc Nephrol. 21(11):1928–41, 2010 10.1681/ASN.2009121257 20966128 PMC3014007

[16] Alper SL. Molecular physiology and genetics of Na+-independent SLC4 anion exchangers. J Exp Biol. 212(Pt 11):1672–83, 2009 Review. 10.1242/jeb.029454 19448077 PMC2683012

[17] Leviel F, Hübner CA, Houillier P, Morla L, El Moghrabi S, Brideau G, et al The Na+-dependent chloride-bicarbonate exchanger SLC4A8 mediates an electroneutral Na+ reabsorption process in the renal cortical collecting ducts of mice. J Clin Invest. 120:1627–35, 2010 10.1172/JCI40145 20389022 PMC2860930

[18] Eladari D, Chambrey R, Peti-Peterdi J. A new look at electrolyte transport in the distal tubule. Annu Rev Physiol. 74:325–49, 2012 Review. 10.1146/annurev-physiol-020911-153225 21888509 PMC4578689

[19] Xu J, Barone S, Li H, Holiday S, Zahedi K, Soleimani M. Slc26a11, a chloride transporter, localizes with the vacuolar H(+)-ATPase of A-intercalated cells of the kidney. Kidney Int. 80(9):926–37, 2011 10.1038/ki.2011.196 21716257 PMC11709004

[20] Sindić A, Chang MH, Mount DB, Romero MF. Renal physiology of SLC26 anion exchangers. Curr Opin Nephrol Hypertens. 16(5):484–90, 2007 Review. 17693766 10.1097/MNH.0b013e3282e7d7d0

[21] Soleimani M. SLC26 Cl-/HCO3- exchangers in the kidney: roles in health and disease. Kidney Int. 84(4):657–66, 2013 Review. 10.1038/ki.2013.138 23636174 PMC10947778

[22] Verlander JW, Hassell KA, Royaux IE, Glapion DM, Wang ME, Everett LA, et al Deoxycorticosterone upregulates PDS (Slc26a4) in mouse kidney: role of pendrin in mineralocorticoid-induced hypertension. Hypertension. 42:356–62, 2003 12925556 10.1161/01.HYP.0000088321.67254.B7

[23] Amlal H, Petrovic S, Xu J, Wang Z, Sun X, Barone S, et al Deletion of the anion exchanger Slc26a4 (pendrin) decreases apical Cl(-)/HCO3(-) exchanger activity and impairs bicarbonate secretion in kidney collecting duct. Am J Physiol Cell Physiol. 299:C33–41, 2010 10.1152/ajpcell.00033.2010 20375274 PMC2904254

[24] Schultheis PJ, Lorenz JN, Meneton P, Nieman ML, Riddle TM, Flagella M, et al Phenotype resembling Gitelman's syndrome in mice lacking the apical Na+-Cl- cotransporter of the distal convoluted tubule. J Biol Chem. 273(44):29150–5, 1998 9786924 10.1074/jbc.273.44.29150

[25] Soleimani M, Barone S, Xu J, Shull GE, Siddiqui F, Zahedi K, et al Double knockout of pendrin and Na-Cl cotransporter (NCC) causes severe salt wasting, volume depletion, and renal failure. Proc Natl Acad Sci U S A. 109(33):13368–73, 2012 10.1073/pnas.1202671109 22847418 PMC3421168

[26] Ronzaud C, Loffing-Cueni D, Hausel P, Debonneville A, Malsure SR, Fowler-Jaeger N, et al Renal tubular NEDD4-2 deficiency causes NCC-mediated salt-dependent hypertension. J Clin Invest. 123(2):657–65, 2013 10.1172/JCI61110 23348737 PMC3561795

[27] Xiang Y, Ma B, Yu HM, Li XJ. The protein profile of acetazolamide-treated sera in mice bearing Lewis neoplasm. Life Sci. 75(11):1277–85, 2004 15234186 10.1016/j.lfs.2003.12.032

[28] Amlal H, Ledoussal C, Sheriff S, Shull GE, Soleimani M. Downregulation of renal AQP2 water channel and NKCC2 in mice lacking the apical Na+-H+ exchanger NHE3. J Physiol.;553(Pt 2):511–22, 2003 14500765 10.1113/jphysiol.2003.053363 PMC2343572

[29] Brooks HL, Sorensen AM, Terris J, Schultheis PJ, Lorenz JN, Shull GE, et al Profiling of renal tubule Na+ transporter abundances in NHE3 and NCC null mice using targeted proteomics. J Physiol. 530(Pt 3):359–66, 2001 11158268 10.1111/j.1469-7793.2001.0359k.x PMC2278426

[30] Xu J, Barone S, Brooks MB, Soleimani M. Double knockout of carbonic anhydrase II (CAII) and Na(+)-Cl(-) cotransporter (NCC) causes salt wasting and volume depletion. Cell Physiol Biochem. 32(7):173–83, 2013 10.1159/000356637 24429824 PMC10947769

[31] Unwin RJ, Capasso G. Bartter's and Gitelman's syndromes: their relationship to the actions of loop and thiazide diuretics. Curr Opin Pharmacol. 6(2):208–13, 2006 Review. 16490401 10.1016/j.coph.2006.01.002

[32] Arroyo JP, Kahle KT, Gamba G. The SLC12 family of electroneutral cation-coupled chloride cotransporters. Mol Aspects Med. 34(2–3):288–98, 2013 Review. 10.1016/j.mam.2012.05.002 23506871

[33] Wang W, Shen J, Cui Y, Jiang J, Chen S, Peng J, et al Impaired sodium excretion and salt-sensitive hypertension in corin-deficient mice. Kidney Int. 82(1):26–33, 2012 10.1038/ki.2012.41 22418978 PMC3376235

[34] Vallet M, Picard N, Loffing-Cueni D, Fysekidis M, Bloch-Faure M, Deschênes G, et al Pendrin regulation in mouse kidney primarily is chloride-dependent. J Am Soc Nephrol. 17(8):2153–63, 2006 16825334 10.1681/ASN.2005101054

[35] Wall SM, Kim YH, Stanley L, Glapion DM, Everett LA, Green ED, et al NaCl restriction upregulates renal Slc26a4 through subcellular redistribution: role in Cl- conservation. Hypertension. 44:982–7, 2004 15477386 10.1161/01.HYP.0000145863.96091.89

[36] Purkerson JM, Schwartz GJ. The role of carbonic anhydrases in renal physiology. Kidney Int. 71(2):103–15, 2007 Review. 17164835 10.1038/sj.ki.5002020

[37] Nicoletta JA, Schwartz GJ. Distal renal tubular acidosis. Curr Opin Pediatr. 16(2):194–8, 2004, Review. 15021201 10.1097/00008480-200404000-00014

[38] Breton S, Alper SL, Gluck SL, Sly WS, Barker JE, Brown D. Depletion of intercalated cells from collecting ducts of carbonic anhydrase II-deficient (CAR2 null) mice. Am J Physiol. 269(6 Pt 2):F761–74, 1995 8594870 10.1152/ajprenal.1995.269.6.F761

[39] Bagnis C, Marshansky V, Breton S, Brown D. Remodeling the cellular profile of collecting ducts by chronic carbonic anhydrase inhibition. Am J Physiol Renal Physiol. 2001 3;280(3):F437–48, 2001. 11181405 10.1152/ajprenal.2001.280.3.F437

[40] Welsh-Bacic D, Nowik M, Kaissling B, Wagner CA. Proliferation of acid-secretory cells in the kidney during adaptive remodelling of the collecting duct. PLoS One.; 6(10):e25240, 2011 10.1371/journal.pone.0025240 22039408 PMC3200326

[41] Sun X, Soleimani M, Petrovic S. Decreased expression of Slc26a4 (Pendrin) and Slc26a7 in the kidneys of carbonic anhydrase II-deficient mice. Cell Physiol Biochem. 21(1–3):95–108, 2008 10.1159/000113751 18209476

[42] Zahedi K, Barone S, Xu J, Soleimani M. Potentiation of the effect of thiazide derivatives by carbonic anhydrase inhibitors: molecular mechanisms and potential clinical implications. PLoS One.;8(11):e79327, 2013 10.1371/journal.pone.0079327 24260196 PMC3832474

